# Anti-CD40 antibody KPL-404 inhibits T cell-mediated activation of B cells from healthy donors and autoimmune patients

**DOI:** 10.1186/s13075-020-02372-z

**Published:** 2021-01-06

**Authors:** John Marken, Sujatha Muralidharan, Natalia V. Giltiay

**Affiliations:** 1 Division of Rheumatology, Department of Medicine, School of Medicine, University of Washington, 750 Republican St, Seattle, WA 98109 USA; 2 Kiniksa Pharmaceuticals Corp, Lexington, MA 02421 USA

**Keywords:** Anti-CD40 mAb, B cell activation, CD40-CD40L co-stimulation, Sjogren’s syndrome (SjS), Systemic lupus erythematosus (SLE), KPL-404

## Abstract

**Background:**

CD40-CD40L is a key co-stimulatory pathway for B cell activation. As such, its blockade can inhibit pathogenic B cell responses in autoimmune diseases, such as Sjogren’s syndrome (SjS) and systemic lupus erythematosus (SLE). In this study, we examined the in vitro effects of KPL-404, a humanized anti-CD40 monoclonal antibody (Ab), on primary human B cells derived from either healthy donors (HD) or autoimmune patients and compared them to the effects of G28-5, a partially antagonistic anti-CD40 antibody.

**Methods:**

PBMCs from HD or SjS and SLE patients were cultured in high-density cell cultures in the presence of IgG4 isotype control or anti-CD40 Abs KPL-404 or G28-5. Cells were stimulated with anti-CD3/CD28 cross-linking reagent ImmunoCult (IC) to induce CD40L-CD40-mediated B cell responses. B cell proliferation and activation, measured by dilution of proliferation tracker dye and the upregulation of CD69 and CD86, respectively, were assessed by flow cytometry. Anti-CD40 Ab cell-internalization was examined by imaging flow cytometry. Cytokine release in the PBMC cultures was quantified by bead-based multiplex assay.

**Results:**

KPL-404 binds to CD40 expressed on different subsets of B cells without inducing cell depletion, or B cell proliferation and activation in in vitro culture. Under the same conditions, G28-5 promoted proliferation of and increased CD69 expression on otherwise unstimulated B cells. KPL-404 efficiently blocked the CD40L-CD40-mediated activation of B cells from HD at concentrations between 1 and 10 μg/ml. Treatment with KPL-404 alone did not promote cytokine production and blocked the production of IFNβ in healthy PBMC cultures. KPL-404 efficiently blocked CD40L-CD40-mediated activation of B cells from patients with SjS and SLE, without affecting their anti-IgM responses or affecting their cytokine production. Consistent with the differences of their effects on B cell responses, KPL-404 was not internalized by cells, whereas G28-5 showed partial internalization upon CD40 binding.

**Conclusions:**

Anti-CD40 mAb KPL-404 showed purely antagonistic effects on B cells and total PBMCs. KPL-404 inhibited CD40L-CD40-mediated B cell activation in PBMC cultures from both healthy controls and autoimmune patients. These data support the therapeutic potential of CD40 targeting by KPL-404 Ab for inhibiting B cell responses in SjS and SLE.

## Background

The CD40-CD40L pathway is a key co-stimulatory pathway for driving T cell-dependent B cell activation and humoral immune responses. CD40 engagement on B cells promotes B cell activation and proliferation and drives the formation of germinal centers (GC) where antibody isotype switching and affinity maturation occurs, leading to the generation of memory B cells and long-lived plasma cells. Furthermore, CD40-CD40L crosstalk promotes antigen (Ag) presentation and conditions Ag-presenting cells to prime robust T cell responses [1–4].

Consistent with its role in humoral immunity, the CD40-CD40L pathway has been implicated in the pathogenesis of several autoimmune diseases, known to be driven by the production of pathogenic autoantibodies. For example, CD40 polymorphisms, linked to increased CD40 proteins levels, are associated with a higher risk of developing systemic lupus erythematosus (SLE) and Graves’ disease [5–8]. Furthermore, CD40-CD40L interactions have been implicated in the formation of ectopic GCs in salivary glands in Sjogren’s syndrome (SjS) and thyroid gland in Graves’ disease, and the generation of antibody-producing plasma cells [9–11]. The inhibition of CD40-CD40L pathway improves disease pathology in mouse models of lupus by reducing B cell activation, T follicular helper cell (T_FH_) cell expansion, and the development of glomerulonephritis [12, 13]. The inhibition of CD40-CD40L pathway also inhibits autoimmune pathology in models of SjS, autoimmune thyroid disease, and experimental autoimmune uveoretinitis [14, 15].

The use of anti-CD40L antibodies has shown benefits in patients with SLE [16, 17]. However, their use was linked to the development of thromboembolic events due to the engagement of CD40L on platelets with subsequent platelet activation [18]. The use of modified anti-CD40L Abs, such as anti-CD40L pegylated Fab fragments, which do not activate platelets, were further explored for their ability to reduce autoimmunity [19–21]. Recent clinical data, however, suggest that their use may not be efficient in treating SLE [22].

Several anti-CD40 mAbs have been developed, many of which show agonistic, or partial agonistic functions [23, 24]. One such mAb is G28-5, used for the original discovery of CD40 [25, 26], which is described as partially antagonistic. However, the effects of G28-5 on B cell depletion and B cell activation have not been fully evaluated.

Recent studies also describe the identification of antagonistic anti-CD40 Abs, which can prolong allograft survival in models of transplantation and inhibit T cell-induced B cell responses. Several of these Abs are being evaluated in human trials [27–31].

In this current study, we examined the properties of KPL-404, a new immunoglobulin G (IgG)4 anti-CD40 mAb, developed based on a previously described anti-CD40 antibody 2C10, a non-depleting mAb, which was found to have immunosuppressive functions [32]. The fully humanized antibody KPL-404 retains an immunologically silent Fc with a S228P mutation to stabilize the Fab hinge and limit IgG4 Fab-arm exchange, which can occur with human IgG4 antibodies [33–36].

We evaluated KPL-404 binding to human B cells and its effects on B cells from healthy individuals and patients with SjS and SLE and compared its effects to the effects of G28-5.

KPL-404 did not exert any agonistic activity on B cells in vitro and inhibited CD40L-CD40-dependent B cell activation at nanomolar concentrations, providing a basis for further testing of KPL-404 in patients with autoimmune diseases.

## Methods

### KPL-404 binding affinity analysis

Binding kinetics of KPL-404 to recombinant human CD40 molecule (Sino Biologicals) was analyzed using the IBIS MX96 following direct immobilization of Ab onto a Xantec SPMX CMD501 sensor chip. Immobilization was performed via amine coupling—at both 10 μg/mL and 20 μg/mL loading densities—in 10 mM acetate, pH 4.5 using the CFM printer. Analytes were run in a kinetics series of 500 nM, 250 nM, 125 nM, and 62.5 nM. The data were analyzed using a 1:1 bimolecular interaction model to calculate association and dissociation rates. The KD [equilibrium dissociation constant = Koff (dissociation rate)/Kon (association rate)] was calculated using Carterra’s KIT analysis software.

### Study subjects

Fresh whole blood was obtained from 8 healthy donors (HD), 8 SLE, and 7 SjS patients under an approved IRB protocol. Patients were recruited from the University of Washington Medical Center. For SLE, disease activity was measured using the SLE Disease Activity Index 2000 (SLEDAI-2K). Patients treated with biologics within the last 6 months or suspected of having acute infections were excluded from the analysis. HD with no history of autoimmune diseases or current infections were used. Additional information about the SjS and SLE study subjects included in the analysis is shown in Supplemental Table 1.

### Cell isolation

PBMCs were isolated as buffy coat from density gradient centrifugation at 1200×*g* using Ficoll-Paque (Sigma) and sepMate-50 centrifuge tubes (StemCell Technologies). PBMCs were washed twice in PBS supplemented with 2% FBS by centrifugation at 300×*g* and suspended in ImmunoCult™-XF T Cell Expansion Medium (StemCell Technologies). This media was used for all cell stimulations and cell cultures.

### Cell stimulation

PBMCs were cultured at 0.5 to 1 million cells/well in 96-well plates at 37 °C in 100 μl of ImmunoCult™-XF T media (high-density PMBC cell culture). Cells were incubated with IgG4 control, antibody KPL-404, or antibody G28-5 at concentration 10 μg/ml, and (without Ab pre-incubation) either left untreated (media control), or stimulated with, anti-CD3/CD28 ImmunoCult (IC) 2.5 μl/100 ml, or 10 μg/ml AffiniPure F(ab′)_2_ fragment goat anti-human IgM (H+L) (Jackson Immunoresearch) for 16–18 h for assessing cell activation. Cell survival and proliferation experiments were performed with 24 h and 5-day cultures with the same Ab concentrations. At the end of the incubation periods, supernatant was retained for cytokine analysis and cells analyzed by flow cytometry. Titration experiments were performed with varying concentrations (20 to 0.01 μg/ml) of IgG4 isotype or anti-CD40 antibody in the same cell stimulation model, with antibody ranges chosen based on previous studies [32].

### Flow cytometry

The PBMCs from the 16 to 18-h incubations were harvested and stained on ice in staining media (PBS with 2% FBS and 0.02% sodium Azide) with the following antibodies: Fc block (anti-CD32), Brilliant Violet 421™ anti-human CD40 Ligand, Brilliant Violet 605 anti-human CD4, Alexa Fluor® 488 anti-human CD19, PE-Cy7 anti-human CD69, and Alexa Fluor® 647 anti-human CD86 (BioLegend). The cells were washed twice by centrifugation at 350×*g* and stained with the fixable viability dye zombie NIR (BioLegend) in PBS at 1:1000 dilution for 30 min. on ice and then washed again in cell-staining media. Legendplex ultracomp compensation beads (BioLegend) were stained with 1/10th concentration of the above antibodies. ArC™ Amine compensation beads (Thermofisher) were stained with zombie NIR fixable viability dye. The stained cells and beads were analyzed on a 4-laser Cytoflex flow cytometer (Beckman Coulter). Compensation and cell analysis was performed on FlowJo software (Tree Stars). T and B cells were identified as CD4^+^ or CD19^+^ positive respectively after gating on single, live lymphocytes and further analyzed for the expression of activation markers, CD69, CD86, and CD40L. Fluorescence minus one (FMO) controls negative/negative gates.

### Cell proliferation analysis

PBMCs were washed in PBS and labeled with Tag-It Violet™ cell tracking dye (BioLegend) at 1:1000 dilution in PBS for 30 min at room temperature. The cells were washed in growth media and stimulated and cultured as above for 5 days and analyzed by flow cytometry. Cell proliferation was quantified using FlowJo software by the dilution of cell Tag-It Violet™ fluorescence intensity. Proliferation was reported as the Division Index (total number of divisions/total number of cells).

### Cytokine analysis

Cell supernatants were analyzed using Legendplex 13 x Viral Response Panel (IL-1β, IL-6, IL-8, IL-10, IL-12p70, IFN-α2, IFN-β, IFN-λ1, IFN-λ2/3, IFN-γ, TNF-α, IP-10, GM-CSF) (BioLegend) per manufacturers protocol. Twenty-five microliters of cell supernatant was incubated with 25 μl incubation buffer and 25 μl bead mix per well for 2 h. at room temperature, in a V-bottom 96-micro well plate supplied with the Legendplex kit while shaking at 800 rpm on a short-radius platform shaker. The beads were centrifuged at 300×*g* for 5 min and re-suspended in 150 ml wash buffer supplied with the kit and centrifuged again. The washed beads were stained with 25 μl detection antibody mix for 1 h followed by the addition of 25 μl of PE-streptavidin mix and incubated for an additional 30 min. The beads were washed once more as above. Standard curves for each cytokine was performed by staining beads with dilutions of the Legendplex kit supplied standard mix. Beads and cells were analyzed on a Cytoflex flow cytometer and analyzed using FlowJo software. Individual bead sets for each cytokine were gated by size and APC fluorescence. Median fluorescence intensity (MFI) for each bead population was obtained. Cytokine concentrations were determined by 4-parameter curve fitting of the standard curve results using Graphpad Prism software.

### Antibody labeling

Anti-CD40 antibodies were fluorescently labeled using the Alexa Fluor-647 (AL647) Microscale Protein Labeling Kit (ThermoFisher) according to the manufacturer’s instructions to a target degree of labeling of 20 x. Briefly, 100 μg of antibody (PBS, 0.1 M Sodium Bicarbonate) were incubated with 1 μg of 7.94 M labeling reagent for 15 min at room temperature. Labeled antibody was separated from free label using micro-spin columns with 800 μl G25 resin by centrifugation for 60 s at 16,000×*g*.

### Ab internalization and imaging flow cytometry

The PBMCs were first washed with PBS, incubated for 20 min on ice with FVD, then washed in RPMI and stained on ice for 40 min in staining media (RPMI with 2% FBS and Fc block) with or without 0.1% Sodium Azide (set 1 and set 2, respectively) with anti-CD19-Alexa Fluor-488, anti-CD40 KPL-404-AL647 or G28-5-AL647, or anti-CD22-AL647 control (1 μg/ml). Cells were washed 2x in RPMI+ 2%FBS and either kept on ice (set 1), or incubated at 37 °C (set 2) for 1 h. Cells were washed 1x with PBS, fixed in 1% formalin, kept on ice, and further analyzed. Internalization analysis was performed using Amnis® Imaging Flow Cytometer (Image Stream X Mark II) and data was analyzed using IDEAS® Software. RMS gradient gating was used to define focused cell images. After gating on singlets and live cells, B cells were defined based on CD19 positivity. Two to 5 million PBMCs were used to obtain sufficient cell density to run 50,000 events and obtain 400–500 images of CD19^+^ cells which were then analyzed for anti-CD40 and anti-CD22 binding and internalization. Masks were created in the IDEAS software based on bright field images (Ch04) for the cell interior and for the cell membrane area (whole cell mask minus internal erode mask). MFI for the APC channel (Ch05) within the internal mask was compared with that for the cell membrane mask. Internalization was expressed as a ratio of the internal mask MFI and membrane mask MFI.

### Statistical analysis

MFI for activation markers CD69, CD86, CD40L on CD4^+^ T cell populations and CD19^+^ B cell populations were normalized to unstimulated IgG4 controls for each individual donor. One-way ANOVA on log-transformed MFI with matched mixed-effects comparisons and non-parametric unmatched multi-comparison analysis was performed using the Kruskal-Wallace test with Dunn’s multiple comparison tests. Additional tests used for normalized data were Wilcoxon signed rank test to test for significance from IgG4 unstimulated control set to the value of 1.0. Two-way ANOVA was used to compare cell proliferation index data. Internal/external image mask ratios were compared by one-way ANOVA. Cytokine concentrations were normalized against the unstimulated IgG4 control for each donor and each cytokine. One and two-way ANOVA, or Friedman’s non-parametric repeated measures comparisons test were used to determine statistical significance. Statistical analyses were performed using GraphPad Prism software (GraphPad Software, Inc.)

## Results

### KPL-404 shows high affinity for human CD40 and binds to different subsets of B cells

**Fig. 1:**
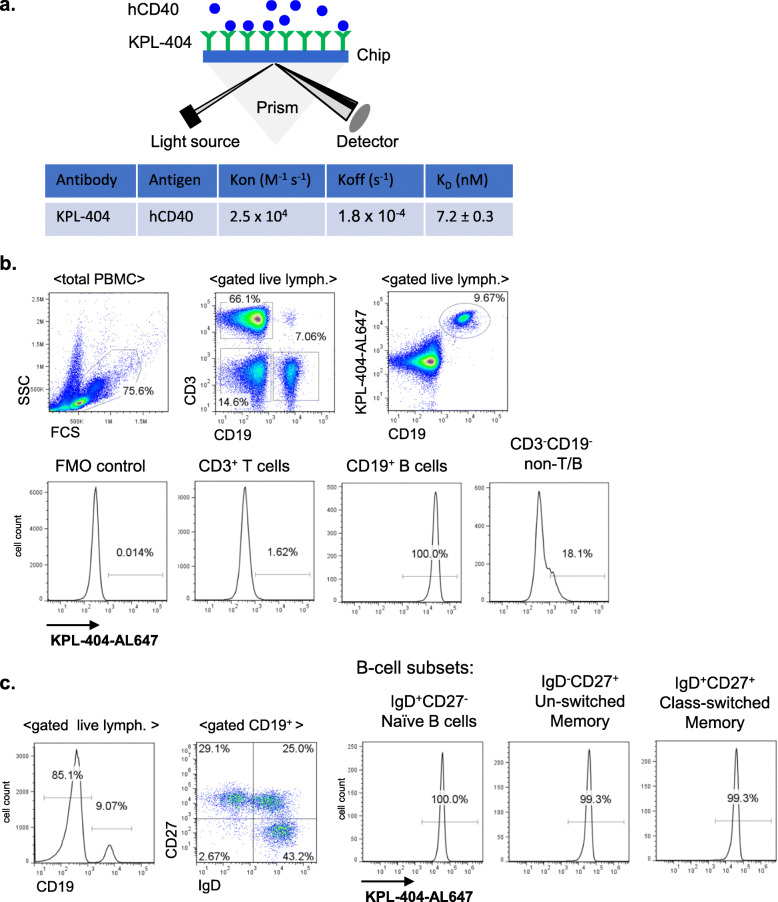
KPL-404 affinity and binding to primary B cells. **a** Binding kinetics of KPL-404 to recombinant human CD40 molecule was analyzed following direct immobilization of KPL-404 onto a sensor chip. CD40 analyte was flowed over the immobilized KPL-404. The KD [equilibrium dissociation constant = Koff (dissociation rate)/Kon (association rate)] was calculated by analyzing the change in reflection of incident light on the chip as a result of KPL-404 and CD40 interaction. **b** PBMCs were stained with cell lineage markers and AL647-labeled KPL-404 Ab. Representative data from one HD. Histograms show the percentage KPL-404-AL647^+^ cells within different cell subsets, including CD19^+^ (B cells), CD3^+^ (T cells), and CD19^−^CD3^−^ (non-T/B). **c** B cell subsets within CD19^+^ PBMCs were defined based on the expression of IgD and CD27. Histograms show the percentage KPL-404^+^ cells within different subsets, including naïve (IgD^+^CD27^−^), un-switched memory (IgD^+^CD27^+^), and class-switched memory (IgD^−^CD27^+^) B cells. Data are representative of three independent experiments, using PBMCs from different donors

Affinity and kinetics of KPL-404 for human CD40 were determined by surface plasmon resonance (SPR) analysis by analyzing the change in reflection of incident light on the chip as a result of KPL-404 and CD40 interaction. KPL-404 was immobilized on the chip and recombinant human CD40 protein was flowed as the analyte. The equilibrium dissociation constant (*K*_D_) was estimated as 7.2 nM, with an association rate constant (Kon) of 2.5 × 104 M^−1^ s^−1^, and a dissociation rate constant (Koff) of 1.8 × 10–4 s^−1^ (Fig. 1a).

To further test if KPL-404 binds CD40 expressed on primary human B cells, we stained healthy PBMCs with AL647-labeled KPL-404 (KPL-404-AL647) and assessed its binding to total CD19^+^ B cells, CD3^+^ T cells, and CD19^−^CD3^−^ (non-T/B cell) populations (Fig. 1b). As expected, KPL-404 showed no binding to T cells, while KPL-404 bound to all CD19^+^ cells KPL-404 bound to about 15–20% of non-T/B cells, majority of which were CD14^+^ monocyte (Mo) cells, also known to express CD40 (Fig. S1A).

Next, we evaluated KPL-404 binding within CD19^+^ peripheral B cell subsets, including naïve, un-switched and class-switched memory B cells, defined based on their surface IgD and CD27 expression (Fig. 1c). KPL-404 show binding to all B cells, with no significant differences between different subsets. Since GC B cells are not present in PBMCs, we also analyzed KPL-404 binding in CD19^+^ tonsillar B cells. Imaging flow cytometry analysis showed KPL-404-AL647 surface staining in both IgD^+^CD27^−^ (naïve B) and IgD^+^CD27^−^ cells (which consist of a mixed population of memory B cells and GC B cells) (Fig. S1B).

Together, these data show that KPL-404 binds with high affinity to CD40 expressed on different subsets of B cells, including naïve, memory, and GCs and, therefore, may affect their responses.

### KPL-404 does not deplete B cells in PBMC cultures

**Fig. 2:**
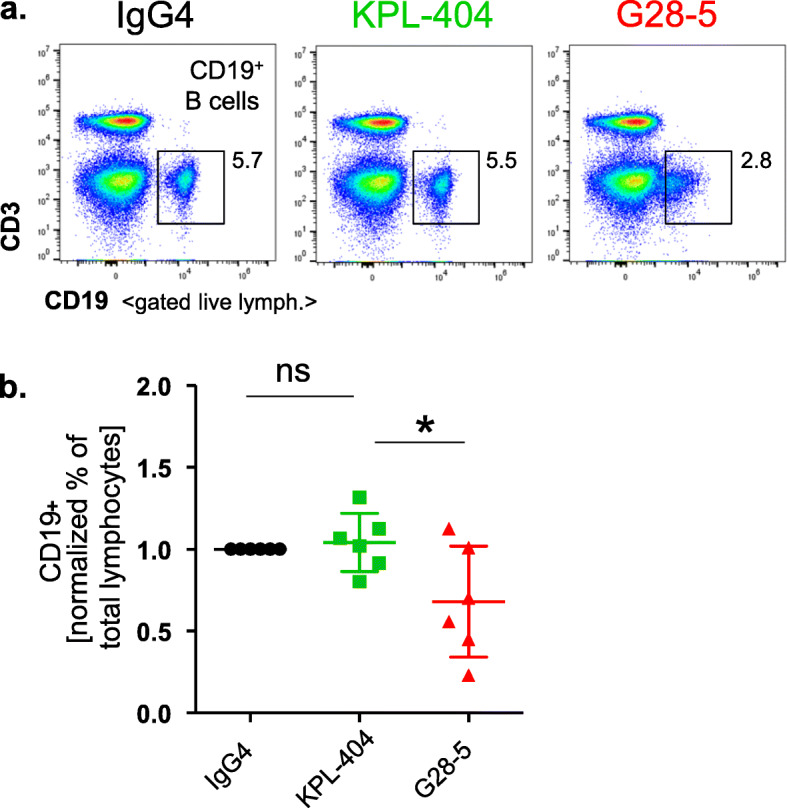
KPL-404 does not deplete B cells in culture. PBMCs were cultured in the presence of 10 μg/ml IgG4 isotype control or anti-CD40 Abs KPL-404, or G28-5 (16–18 h of cell culture). **a** Representative flow data showing the percentage of CD19^+^ B cells of gated live lymphocytes. **b** Summary data from 6 individual HD showing the percentage of live CD19^+^ cells in the lymphocyte population. B cell frequencies for each donor are normalized to IgG4 control. The bars represent mean and standard deviation (SD). *p < 0.05, determined by unmatched ANOVA analysis with multiple comparisons to control IgG4

We next evaluated the effects of KPL-404 in in vitro PBMC cell cultures from HD and compared it to the effects of anti-CD40 mAb - G28-5, known to be a partial CD40 antagonist [26]. The percentage and number of CD19^+^ B cells in 16–18-h PBMC cultures were not affected by KPL-404, indicating a lack of B cell depletion (Fig. 2a, b). Similarly, we found no increase in cell death or any significant changes in CD19^+^ B cell frequencies relative to IgG4 isotype control samples after 3–5 days of culture. In comparison, the percentage of CD19^+^ B cells were significantly reduced in the presence of anti-CD40 mAb, G28-5, suggesting that, in contrast to KPL-404, G28-5 depletes B cells or, it might affect the expression of CD19 upon its binding to CD40 (Fig. 2a, b).

### KPL-404 inhibits T cell-mediated B cell proliferation

Next, we assessed the effects of KPL-404 on B cell proliferation. In this set of experiments, we labeled PBMCs with cell proliferation tracker dye and cultured them for 5 days in the presence of IgG4 (isotype control Ab for KPL-404) or anti-CD40 Abs, KPL-404, and G28-5, and then measured B cell proliferation. We left cells untreated (media control) or stimulated them with anti-CD3/CD28 cross-linking reagent IC, which, when added to HD PBMC cultures, promoted T cell activation, as evidenced by the increase in CD40L and CD69 expression 16–18 h after stimulation as well the increase in T cell proliferation after 5 days in culture (Fig. S2A-D).

**Fig. 3:**
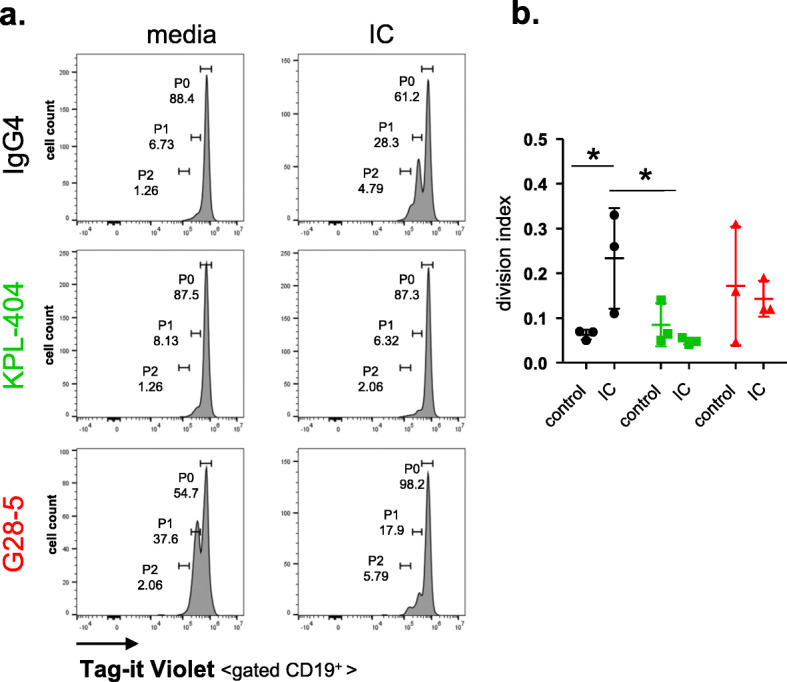
Effects of anti-CD40 antibodies KPL-404 and G28-5 on B cell proliferation. PBMCs were labeled with a cell proliferation tracker dye (Tag-it Violet) and cultured for 5 days in the presence of 10 μg/ml IgG4 isotype control Ab or anti-CD40 Abs—KPL-404 and G28-5. Cells were left untreated (media control) or stimulated with anti-CD3/CD28 cross-linking reagent ImmunoCult (IC). Levels of CD19^+^ B cell proliferation were measured based on Tag-it Violet dilution. **a** Flow cytometry data from one representative experiment. Gates depict non-proliferating cells (gate P0) or Tag-it Violet dim (proliferating) cells (gates P1-P2). **b** Cumulative data from 3 individual donors. B cell proliferation is presented as division Index (total number of divisions/total number of cells) for each sample. The bars represent mean and standard deviation (SD). *p < 0.05, determined by two-way ANOVA with multiple comparisons test

Unstimulated (media control) samples showed no increase in B cell proliferation in the presence of IgG4 isotype or KPL-404 alone. In contrast, G28-5 alone induced some B cell proliferation (observed in two of three independent experiments, using different donors) (Fig. 3a, b).

The activation of T cells with IC-induced B cell proliferation via CD40L-CD40 interactions, as illustrated by the inhibition of observed B cell proliferation in the presence of KPL-404. The degree of IC-induced proliferation was highly variable, likely in part due to different resting state and responses of the individual subject sources used in each experiment. In the same experimental conditions, G28-5 showed only a partial inhibitory effect on B cell proliferation (Fig. 3a, b). The KPL-404 effects on cell proliferation were limited to B cells and did not affect IC-induced T cell proliferation (Fig. S2E).

### Effects of KPL-404 and G28-5 on B cell activation assessed by the expression of the activation markers CD69 and CD86

We also explored the effects of KPL-404 and G28-5 on T cell-mediated B cell activation in 16–18-h HD PBMC cultures. We stimulated cells with IC in the presence of IgG isotype control Ab, KPL-404, or G28-5 and then assessed the expression of B cell activation markers—CD69 and CD86. For control purposes, we also stimulated cells with F(ab′)2 anti-human IgM (anti-IgM), which induces direct B cell activation by surface B cell receptor (BCR) cross-linking.

**Fig. 4:**
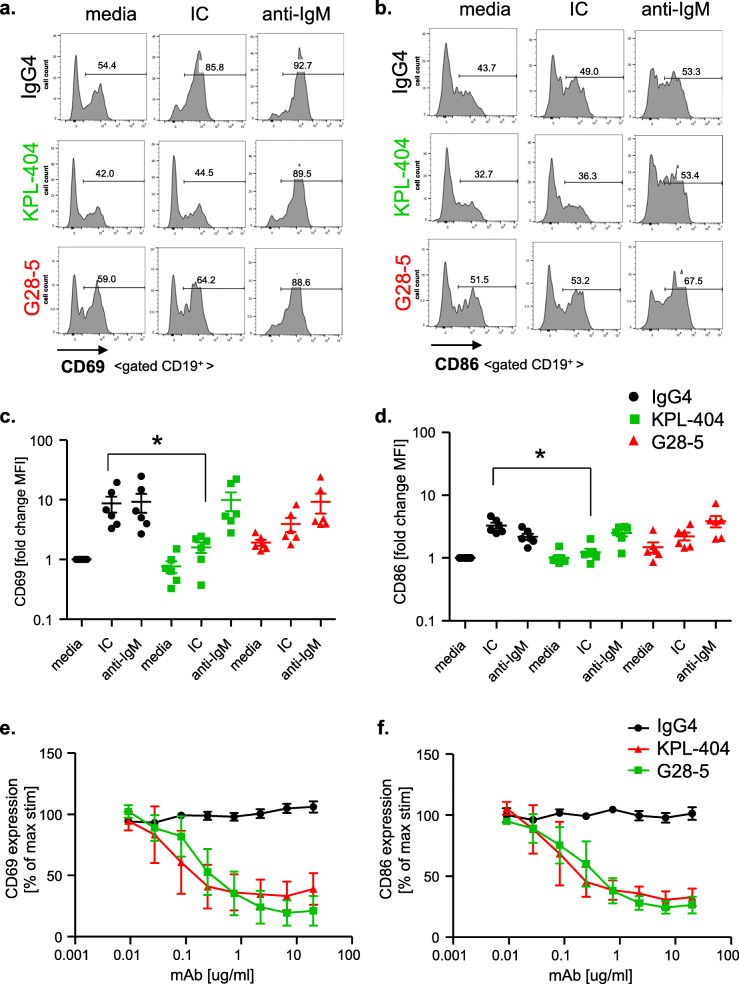
Effect of Anti-CD40 Abs KPL-404 and G28-5 on T cell-dependent B cell activation in healthy donors. PBMCs were cultured in the presence of 10 μg/ml IgG4 isotype control or anti-CD40 Abs KPL-404, or G28-5 (16–18 h of cell culture). Cells were left unstimulated (media control) or stimulated with CD3/CD28 cross-linker IC or F(ab′)_2_ goat anti-human IgM (anti-IgM). **a**, **b** Flow cytometry data from one representative experiment, showing CD69 (**a**) and CD86 (**b**) expression on gated live CD19^+^ B cells. **c**, **d** Cumulative data, depicting the expression of CD69 (**c**) and CD86 (**d**) in six individual HD donors. Data are expressed as fold change MFI over IgG4-teated, media controls. Data were analyzed using one-way ANOVA on log-transformed data with matched mixed-effects modeling for multiple comparisons tests of significance between different conditions; *p < 0.05. Only comparisons between IgG4 vs KPL-404 of IC-stimulated samples are shown. Complete statistical analysis of the data is presented as Supplemental Fig. S3. **e**, **f** PBMCs were stimulated with IC in the presence of varying concentrations (20 to 0.01 μg/ml) of IgG4 isotype antibody control, KPL-404, or G28-5 anti-CD40 antibodies. Graphs show the expression of CD69 (**e**) and CD86 (**f**) on gated CD19^+^ B cells, relative to isotype control (100% max. stimulation) for each Ab. Cumulative data from three independent experiments using different donors

As shown in Fig. 4, CD69 and CD86 expression on B cells was upregulated in response to both IC and anti-IgM. Similar to the results on B cell proliferation, CD69 and CD86 upregulation was blocked in the presence of KPL-404, while G28-5 showed only partial inhibitory effects. The combined analysis of B cell activation in 6 individual donors showed a statistically significant effect of KPL-404 in blocking CD69 and CD86 upregulation in response to IC, while CD69 and CD86 upregulation in response to anti-IgM were not affected by KPL-404 (Fig. 4a–d and Fig. S3). We also found that KPL-404 also blocked the upregulation of other B cell activation markers -CD23 and CD95 while G28-5 promoted the expression of CD23 in unstimulated cells and only partially blocked CD95 upregulation in IC-stimulated samples (Fig. S4).

Titration experiments showed that KPL-404 most efficiently inhibits B cell activation, measured by CD69 and CD86 upregulation, at a concentration between 0.247 and 20 μg/ml (Fig. 4e, f). Titration curves revealed further differences between KPL-404 and G28-5 with respect to their ability to inhibit CD69 expression. At higher concentrations, KPL-404 appeared to more effectively inhibit IC-induced B cell CD69 expression, although the difference did not reach statistical significance.

Combined, our data show that, upon its binding to CD40, KPL-404 alone does not induce B cell proliferation, activation, or depletion. While KPL-404 lacked any agonistic effects, G28-5 displayed partial agonistic functions on B cells. Furthermore, KPL-404 efficiently inhibited IC-induced/T cell-mediated B cell proliferation and blocked the expression of CD40L-CD40-induced key B cell activation markers.

### KPL-404 does not internalize upon binding to CD40 on primary B cells

**Fig. 5:**
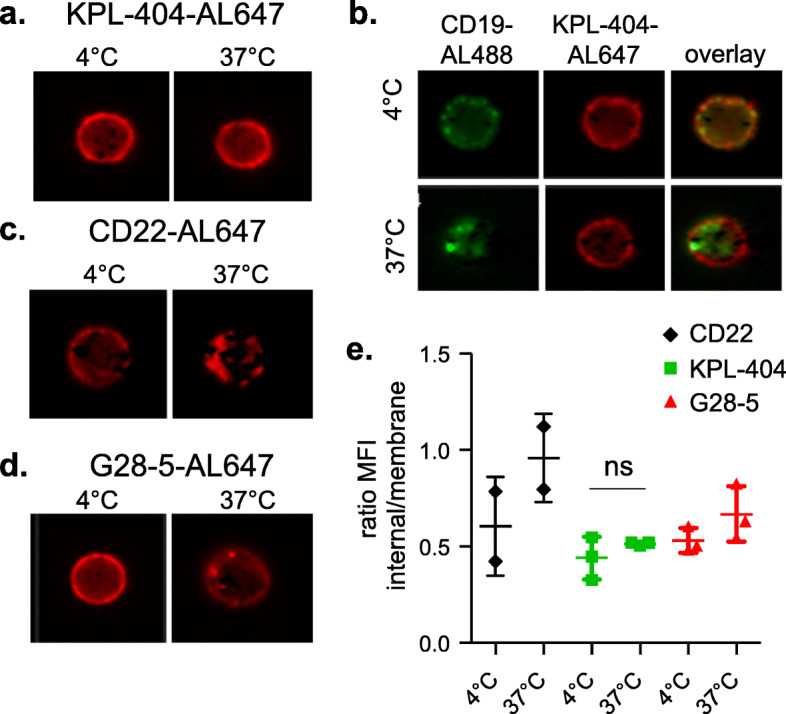
KPL-404 is not internalized upon binding to CD40 on primary B cells. HD PBMCs were stained with FVD and anti-CD19-Alexa Fluor 488, anti-CD40 KPL-404-AL647 or G28-5-AL647, or anti-CD22-AL647 control (1 μg/ml) and analyzed by imaging flow cytometry. Gated live CD19^+^ B cells (gating strategy is presented in Fig. S6) comprising 400–500 B cell images were analyzed for Ab internalization at 4 °C, 0.1% sodium azide (non-permissive conditions) or after incubation at 37 °C for 1 h (permissive conditions). **a**–**d** Representative images of B cell analysis. (**a**) KPL-404-AL647 binding and internalization; (**b**) CD19-Alexa Fluor 488 and KPL-404-AL647 binding and internalization, showing CD19 and KPL-404 signal overlay; (**c**) anti-CD22-AL647 binding and internalization; and (**d**) G28-25-AL647 binding and internalization under non-permissive (4 °C) or permissive conditions (37 °C). **e** Cumulative data of three independent experiments. Graph depicts the ratios of the internal mask MFI and membrane mask MFI as a measure of internalization

CD40 is internalized as part of the signaling process after its binding to CD40L [37]. Previous studies have shown that CD40L and some anti-CD40 Abs are internalized by CD40-expressing cells [27, 38]. Since KPL-404 and G28-5 showed different effects on B cells, next we examined if KPL-404 is internalized upon binding to CD40 on primary human B cells. We incubated PBMCs from HD with fluorescently tagged Abs KPL-404, and G28-5 Abs and then assessed CD40 binding and internalization in B cells at either 4 °C (non-permissive conditions), or after 1 h incubation at 37 °C (permissive conditions). We used CD19 and CD22 as positive controls for the Ab internalization. Imaging results (gating strategy used for the imaging flow cytometry analysis is shown in Fig. S5) show a uniform binding of KPL-404 to the surface of CD19^+^ B cells with no visible internalization at either 4 °C or 37 °C (Fig. 5a). KPL-404 internalization was also not visible after longer incubation time (2 h at 37 °C) (data not shown). As expected, under the same permissive conditions (after 1 h of incubation), both CD19 and CD22 were efficiently internalized (Fig. 5b, c). Unlike KPL-404, G28-5 showed pixelated internal signal after incubation at 37 °C, consistent with its internalization (Fig. 5d); however, quantitatively, the magnitude of the change in internalized fluorescence for G28-5 was much weaker as compared to the CD19 or CD22 signal. Summary data from three independent experiments, using the ratio between the internal mask MFI and membrane MFI for each staining as a measure of Ab internalization, showed no significant increase in the ratios for KPL-404; ratios for G28-5 showed some increase, which however did not reach statistical significance (Fig. 5e).

Overall, these results show that, in the conditions tested, KPL-404 binds to CD40 on the B cell surface without internalizing, whereas G28-5 may partly internalize. These differences are likely to contribute to the observed effects of KPL-404 and G28-5 on B cells.

### KPL-404 inhibits T cell-induced B cell activation in PBMC cultures derived from SjS and SLE patients

We have shown that KPL-404 inhibits IC-induced B cell activation in PBMC cultures derived from HD. Next, we explored if KPL-404 can also inhibit T cell-mediated B cell responses, using PBMCs from patients with autoimmune rheumatic diseases—SjS and SLE, known to be associated with T and B cell hyper-activation and GCs formation. We performed functional studies, similar to those described above using PBMCs from seven patients diagnosed with SjS and eleven patients with SLE (Suppl. Table 1).

**Fig. 6:**
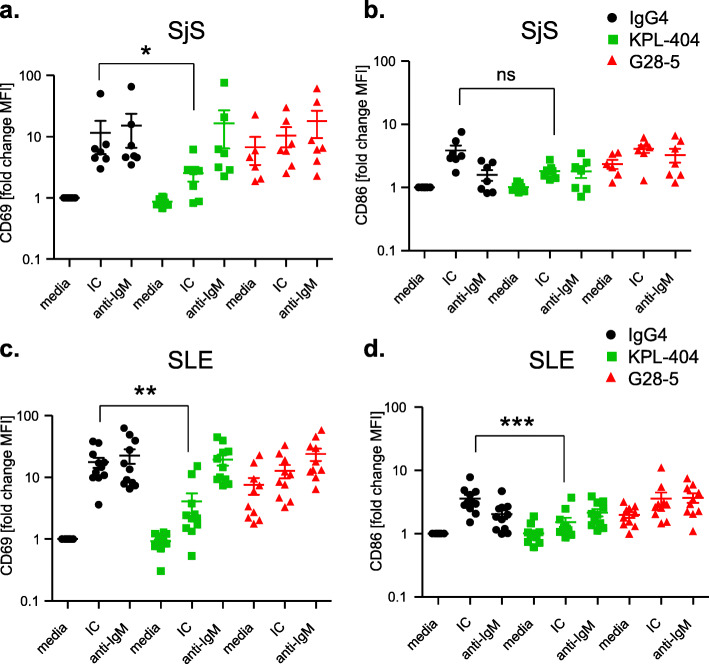
KPL-404 blocks T cell-induced B cell activation in PBMC cultures from SjS and SLE patients. PBMCs were cultured in the presence of 10 μg/ml IgG4 isotype control or anti-CD40 Abs KPL-404 or G28-5 (16–18 h of cell culture). Cells were left unstimulated (media control) or stimulated with CD3/CD28 cross-linker IC, or F(ab′)_2_ goat anti-human IgM (anti-IgM) and B cell activation was assessed by the expression of the activation markers CD69 and CD86 on gated live CD19^+^ B cells. Representative flow cytometry data from one SLE donor is presented on Fig. S5. **a**, **b** Cumulative data, depicting the expression of CD69 (**a**) and CD86 (**b**) in seven individual SjS donors. **c**, **d** Cumulative data, depicting the expression of CD69 (**c**) and CD86 (**d**) in eleven individual SLE donors. Data are expressed as fold change MFI over IgG4-teated, media controls. Bars represent mean and standard deviation. Data were analyzed using one-way ANOVA on log-transformed data with matched mixed-effects modeling for multiple comparisons tests of significance between different conditions, *p < 0.05. Only comparisons between IgG4 vs KPL-404 of IC-stimulated samples are shown. Complete statistical analysis of the data is presented in Fig. S5

Similar to the results in HD PBMCs, KPL-404 alone did not increase the expression of cell activation markers CD69 and CD86 on CD19^+^ B cells from SjS or SLE patients (Fig. 6a–d and S6A-C). Furthermore, the IC-induced upregulation of CD69 and CD86 on B cells was inhibited in the presence of KPL-404. Similar to HD cultures, KPL-404 did not affect B cell responses to BCR cross-linking. G28-5 on the other hand promoted an increase in CD69 expression in unstimulated cells, and G28-5 was less efficient in inhibiting IC-induced B cell activation.

These data show that KPL-404 blocks the activation of B cells from autoimmune (SjS and SLE) patients, suggesting its potential to also inhibit CD40-mediated B cell activation in patients in vivo. As expected, we did not observe any stimulation of CD4^+^ T cell CD69 expression over IgG4 control by KPL-404 or G28-5 (data not shown).

### Effects of KPL-404 and G28-5 on cytokine production in PBMC cultures from healthy donors and autoimmune patients

To better predict the possible in vivo effects of KPL-404, we examined the production of key cytokines, produced by CD40-expressing cells using cell culture supernatants collected from IC- or anti-IgM-stimulated PBMCs from HD, SjS, and SLE patients.

**Fig. 7:**
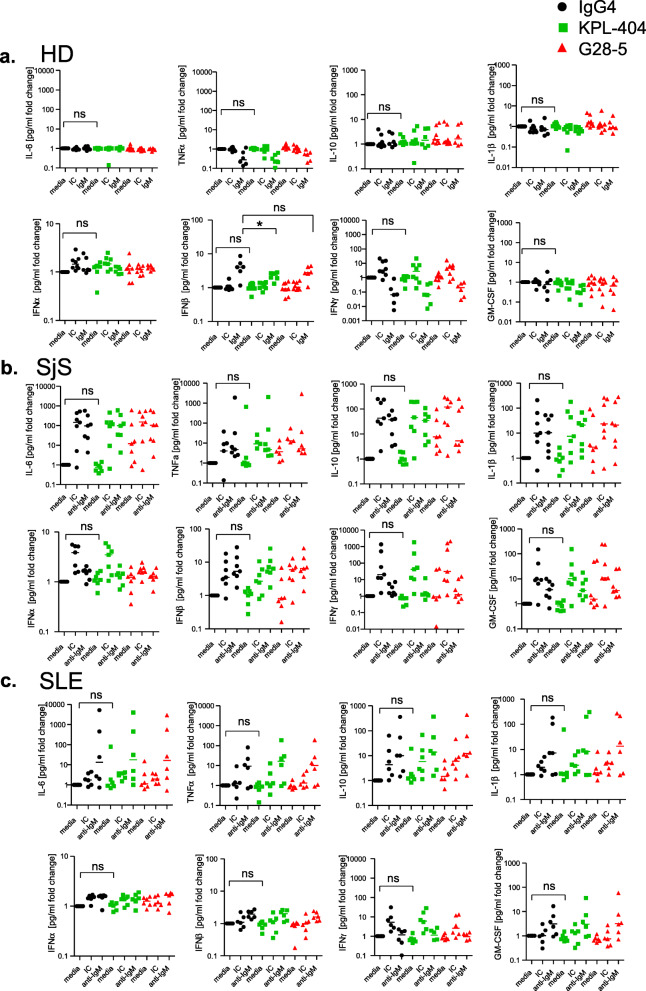
Effects of KPL-404 and G28-5 on cytokine production in PBMC cultures from healthy donors and autoimmune patients. PBMCs were stimulated for 18 h. with media control, IC, or anti-IgM in the presence of either IgG4 isotype control, KPL-404, or G28-5 anti-CD40 antibodies. Supernatants analyzed for production of key inflammatory cytokines. Shown are combined results from six HD (**a**), seven SjS (**b**), and six SLE donors (**c**)**.** Additional data is presented in Fig. S7. The data for each sample and each cytokine were normalized against media controls and is expressed as a fold change. Comparison between IgG4 isotype and KPL-404 of unstimulated samples (media control) are shown (all samples), or differences in IFNβ production in response to anti-IgM stimulation (HDs). Statistical analysis were performed using one-way Friedman multiple comparisons test and one-way ANOVA mixed-effects analysis (for HD due to missing values); *p < 0.05

In HD cell cultures, KPL-404 and G28-5 Abs showed no effect on IL-10 and/or IL-6, known to be produced by B cells. Furthermore, the production of TNFα, IL-10, IL-1β, IFNs, GM-CSF, and IP-10 production was also not affected by KPL-404 Ab alone (Fig. 7a). CD3/CD28 cross-linking by IC stimulation induced an increase in IFNα and IFNγ, which were not promoted or, antagonized by KPL-404 and G28-5 (Fig. 7a). IC also promoted IP-10, which was inhibited by KPL-404 (Fig. S7A). BCR cross-linking (anti-IgM stimulation) led to the reduced production of TNFα and IFNγ but induced IFN-β production in culture, which was inhibited in the presence of KPL-404 and G28-5.

Cytokine responses in SjS and SLE cell cultures were more robust as compared to HD. IC and anti-IgM stimulation in SjS and SLE culture led to an increase in IL-6, TNFα, and IL-10 production as well as an upregulation of IFNα, β and γ, GM-CSF, and IP-10 (Fig. 7b, c and Fig. S7A, which shows fold increase in cytokine production, relative to unstimulated controls, and Fig. S7B, which shows cytokine production in cell cultures, presented as pg/ml). KPL-404 alone did not promote any significant changes cytokine production. Furthermore, KPL-404 did not affect cytokine responses to IC stimulation. Unlike HD samples, KPL-404 showed no effect on IFNβ upregulation in response to anti-IgM stimulation.

These data reveal differences in cytokine responses in response to IC and anti-IgM between HD, SjS, and SLE PBMC cultures. KPL-404 alone did not promote cytokine production and suppressed the production of some inflammatory cytokines in HD, but not SjS, or SLE PBMCs.

## Discussion

CD40-CD40L signaling regulates the interactions between T cells and B cells, and as such it represents an attractive target for suppressing pathogenic B cell responses in autoimmune diseases. In this study, we analyze the in vitro functional properties of KPL-404, a fully humanized IgG4 Ab anti-CD40 monoclonal antibody, which is being developed as a potential therapeutic for patients with autoimmune diseases.

We assessed the effects of KPL-404 in comparison to G28-5, the anti-CD40 mAb used for the discovery of CD40 [25, 26]. Unlike G28-5 and other previously reported anti-CD40 mAb that demonstrate variable levels of agonistic activity [39], KPL-404 showed a purely antagonistic function and did not activate B cells in vitro. KPL-404 also efficiently inhibited T cell-induced B cell activation at low nanomolar concentrations.

SPR analysis showed that KPL-404 has a relativly high binding affinity to human CD40. Specifically, dissociation constant (KD) for KPL-404, determined by SPR analysis, was 7.2 nM. In comparison, G28-5 has a reported affinity of 25 nM (KD) for CD40 [40] which is comparable to the natural affinity of CD40L for CD40 which has been reported to be 10–30 nM (KD) [41]. These data indicate that KPL-404 could bind CD40 more potently than G28-5 or CD40L.

Consistent with data on the expression of CD40 [26], we show that KPL-404 binds to all major subsets of peripheral B cells, including naive and memory B cells, as well as GC B cells, found in human tonsils.

It should be noted that KPL-404 was derived from the previously described anti-CD40 antibody 2C10, a non-depleting mAb which was found to have immunosuppressive functions in allo- and xenotransplantation in non-human primate models [32]. Recent studies have defined the 2C10 binding epitope, located near the membrane-distal tip of CD40. This epitope partially overlaps the CD40L binding site, but, is distinct from the binding sites of previously reported agonistic mAbs [39, 42, 43]. Furthermore, studies suggest that binding of this part of CD40 molecule may stabilize CD40 in an “inactive antiparallel state” and prevent downstream signaling [44].

Characterization of 2C10 activity showed its ability to block binding of CD40L to B cells and to inhibit B cell activation as measured by the decreased expression of CD23 [32, 43]. Consistent with these findings, our results supported KPL-404 antagonistic functions in vitro.

To further explore the effects of KPL-404 on B cell responses, we set up in vitro high-density PBMC cell cultures. The goal of these in vitro experiments was to simulate in vivo conditions where T cells, B cells, and/or other cells that express CD40 and CD40L could interact and stimulate each other. Our data clearly show that, in these settings, KPL-404 lacked any agonistic effects, whereas G28-5 displayed a partial agonistic function on primary B cells. Stimulation with CD28/CD3-cross-linking reagent IC led to the activation of T cells associated with a transient increase in CD40L expression which promoted CD40L-CD40-mediated B cell activation in culture. KPL-404 acted as a pure antagonistic antibody and blocked CD40L-CD40-mediated B cell activation and proliferation; however, it did not affect B cell responses to BCR cross-linking, and did not either potentiate or inhibit B cell responses to other stimuli, including IL-4 or IL-2 (data not shown).

Notably, KPL-404 was able to inhibit IC-induced B cell responses in PBMC cultures obtained from healthy controls and patients with active SjS or SLE disease. This is significant since it suggests that KPL-404 retains its inhibitory properties even if B cells are pre-activated or may have lower activation threshold due to exposure to type I IFNs or other pro-inflammatory cytokines in autoimmune patients.

While KPL-404 inhibited B cell activation in SjS and SLE cell cultures, G28-5 was not efficient in suppressing B cell responses and, similar to HD cultures, seemed to activate B cells even in the absence of other stimuli. As described above, the differences in the effects of G28-5 and KPL-404 we observed are likely due to the fact that they bind to different CD40 epitopes [42]. Our imaging data show that, upon its binding to CD40 on B cells, KPL-404 was not internalized, while G28-5 showed partial internalization. Another antagonistic anti-CD40 Ab CFZ533, which is being evaluated for treatment of autoimmune patients, was shown to internalize upon its binding to CD40 [27], suggesting that Ab internalization alone does not determine whether it will have agonistic or antagonistic properties. These findings could also have implications for the clinical applications of the antibody being tested. Drug internalization could imply a potential sink and affect the pharmacokinetic properties of the molecule in the clinical setting which, owing to its non-internalization, could be an advantage for KPL-404.

KPL-404 has a IgG4 constant region, with a minimum Fc receptor and complement binding, thereby a limited effector function(s) and low cytotoxicity in vivo. In addition, the heavy chain constant region of KPL-404 has a stabilizing proline at hinge position 228 which stabilizes Fab-arm exchange that can occur with human IgG4 antibodies [32, 33, 35, 45]. In should be noted however that our in vitro stimulation experiments for testing KPL-404 effects were performed in serum free media, without additional simultaneous human IgG. Also, in this study,  we did not explore if KPL-404 could undergo Fab-arm exchange, especially since S228P stabilizing mutation might not be sufficient to fully prevent Fab-arm exchange [34]. This is important since therapeutic IgG4 may recombine with endogenous IgG4 that could affect their pharmacokinetics and pharmacodynamics [35]. IgG4 Abs are abundant in patients with Immunoglobulin G4-related disease (IgG4-RD) [46] and have been described in patients with other autoimmune diseases, including Myasthenia gravis and SjS [47, 48]. A further evaluation of a potential half-antibody exchange of KPL-404 with endogenous IgG4 would be required to better predict its in vivo effects in patients.

KPL-404 did not show any significant effects on cytokine responses in cells cultures from HD, except for inhibiting IFNβ and IP-10 production. These inhibitory effects of KPL-404 were not observed in SjS and SLE cell cultures, which, as expected, produced much higher amounts of cytokines (both at basal levels or, in response to IC or anti-IgM stimulation). The effects of KPL-404 on cytokine production should be further investigated in vivo. SjS and SLE cell cultures show some differences in cytokine production, particularly in the production of IL-6 and type I IFNs and GM-CSF, which were more strongly upregulated in SjS cultures in response to IC stimulation. These differences might reflect differences in cell composition and overall cell responsiveness to stimulation and can be further investigated to better understand the pathogenic mechanisms driving these two autoimmune diseases.

## Conclusion

KPL-404, a new humanized non-depleting anti-CD40 monoclonal antibody, binds to B cells and shows purely antagonistic functions on B cell responses. These data provide a strong scientific rationale for testing the clinical utility of KPL-404, and more specifically, its ability to inhibit pathogenic B cell responses in SjS, SLE, and other autoimmune diseases. Our findings also provide new insights into the mode-of-action of antagonistic vs. agonistic anti-CD40 Abs, which could help define their appropriate clinical applications.

## Supplementary Information

**Additional file 1.**

**Additional file 2.**

## Data Availability

The datasets generated during this study are available from the corresponding author upon reasonable request.

## Abbreviations

Ab: Antibody
BCR: B cell receptor
CD40L: CD40 ligand
GC: Germinal center
HD: Healthy donors
IC: ImmunoCult (anti-CD3/CD28 cross-linking reagent)
IFN: Interferon
IL: Interleukin
SjS: Sjögren’s syndrome
SLE: Systemic lupus erythematosus
