# Correction to: Anti-CD40 antibody KPL-404 inhibits T cell-mediated activation of B cells from healthy donors and autoimmune patients

**DOI:** 10.1186/s13075-021-02425-x

**Published:** 2021-01-21

**Authors:** John Marken, Sujatha Muralidharan, Natalia V. Giltiay

**Affiliations:** 1 Division of Rheumatology, Department of Medicine, School of Medicine, University of Washington, 750 Republican St, Seattle, WA 98109 USA; 2 Kiniksa Pharmaceuticals Corp, Lexington, MA 02421 USA

**Correction to: Arthritis Res Ther (2021) 23:5**

**https://doi.org/10.1186/s13075-020-02372-z**

Following publication of the original article [1], a typesetting error was reported. The affiliations for Sujatha Muralidharan and Natalia V. Giltiay are as follows:

- Sujatha Muralidharan: Kiniksa Pharmaceuticals Corp, Lexington, MA 02421, USA
- Natalia V. Giltiay: Division of Rheumatology, Department of Medicine, School of Medicine, University of Washington, 750 Republican St, Seattle, WA 98109, USA

The original article [1] has been corrected.
