# Supplementary material for: Anti-CD40 antibody KPL-404 inhibits T cell-mediated activation of B cells from healthy donors and autoimmune patients
Source: Arthritis Res Ther. 2021 Jan 6;23:5. doi: 10.1186/s13075-020-02372-z (PMC7789801; doi:10.1186/s13075-020-02372-z)
Supplement: Supplementary file 2 — Additional file 2. [file 13075_2020_2372_MOESM2_ESM.pdf]

Fig.S1

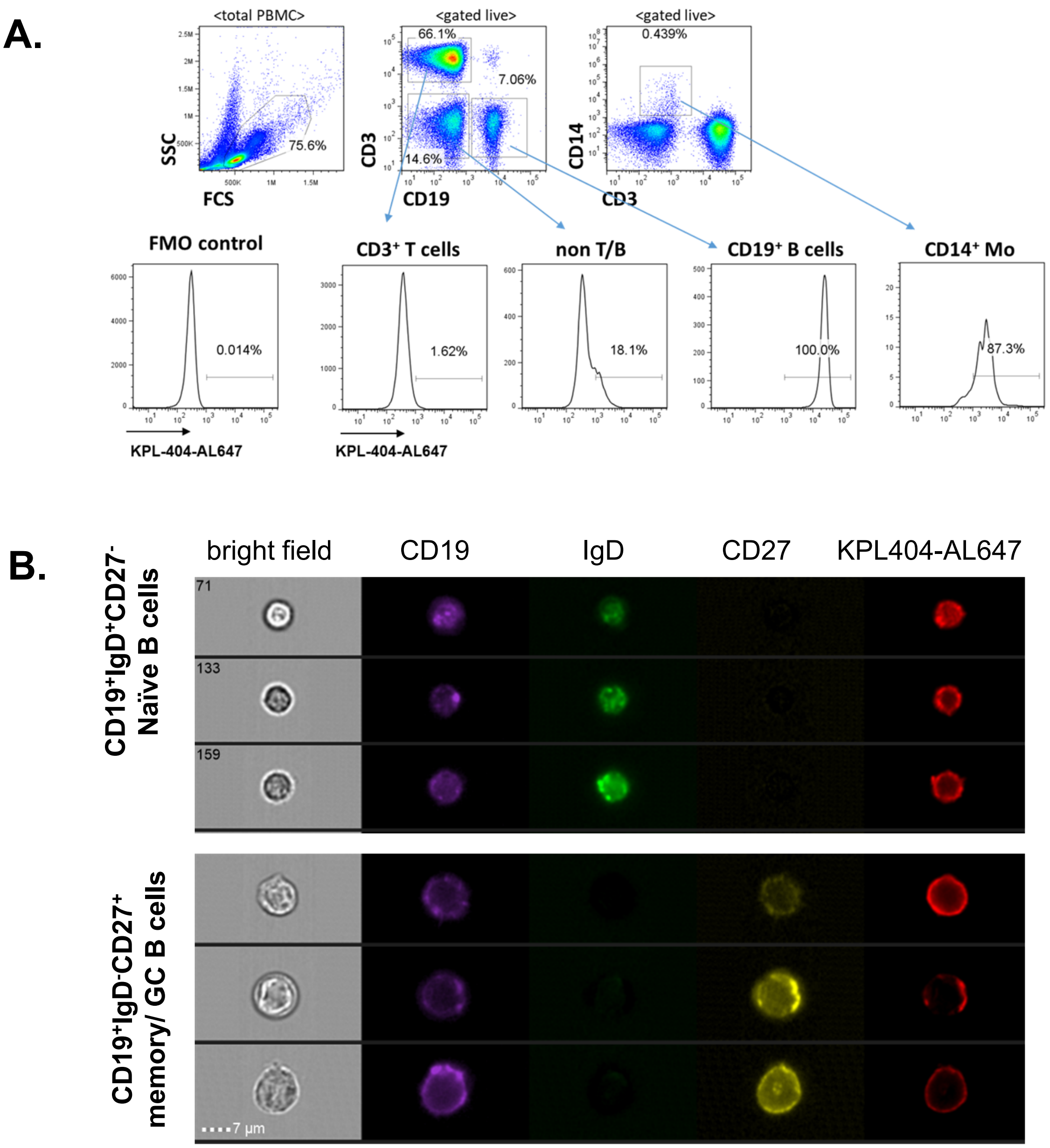

Fig.S1. (A) KPL-404 binding to PBMCs. PBMCs were stained with cell lineage markers and AL647-labeled KPL-404 Ab. Histograms show the percentage KPL-404-AL647^{+} cells within different cell subsets, including CD3^{+} (T cells), CD19^{-}CD3^{-} (non T/B), CD19^{+} (B cells), and CD14^{+} monocytes (Mo). Representative data from one HD. **B) KPL-404 binding to tonsillar B cells.** Tonsillar cells were stained with B cell markers CD19, IgD, CD27, and Alexa-647(AL647)-labeled KPL-404 Ab. Representative imaging flow cytometry data showing KPL-404-AL647 surface staining of IgD^{+}CD27^{-} (naïve B) and IgD^{+}CD27^{-} (memory and GC B cells). Data are representative of two experiments, using cells from different donors.

Fig.S2

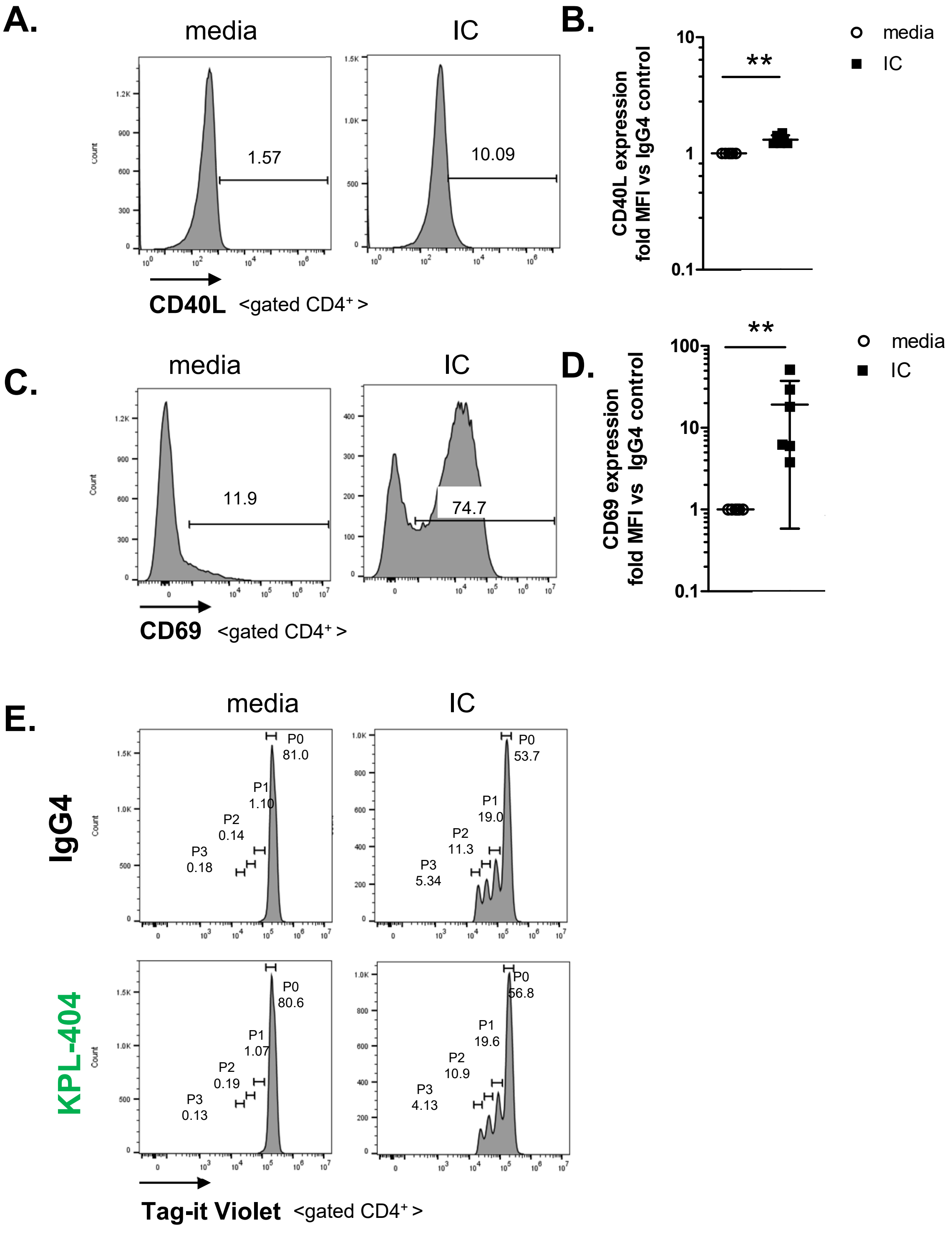

Fig.S2. T cell activation and proliferation in response to CD3/CD28 crosslinking reagent immunocult (IC). (A-D) PBMCs were left untreated (media control), or stimulated with anti-CD3/CD28 cross-linking reagent Immunocult (IC) for 16-18 hours. Representative flow analysis showing the expression of CD40L **(A)** or CD69 **(C)** on gated live CD4⁺ T cell. Cumulative data from six independent experiments, showing upregulation of the expression of CD40L **(B)** or CD69 **(D)** in response to IC stimulation. **p < 0.01, determined by unpaired Student's *t* test. **(E)** PBMCs were labeled with a cell proliferation tracker dye (Tag-it Violet) and cultured for 5 days in the presence of IgG4 isotype control Ab, or KPL-404. Representative flow plots showing increase in T cell proliferation in response to Immunocult (IC) stimulation. Representative data of three independent experiments.

Fig.S3

HD

Treatment	CD69	CD86
IgG4+media vs IgG4+IC	*	**
IgG4+media vs KPL-404+media	ns	ns
IgG4+media vs G28-5+media	*	ns
IgG4+IC vs KPL-404+IC	*	*
IgG4+IC vs G28-5+IC	**	ns
IgG4+media vs IgG4+anti-IgM	*	*
KPL-404+media vs KPL-404+anti-IgM	*	*
G28-5+media vs G28-5+anti-IgM	*	ns
IgG4+IgM vs KPL-404 + anti-IgM	ns	ns
IgG4+IgM vs G28-5+ anti-IgM	ns	ns

Figure S3. Changes in the expression of CD69 and CD86 in healthy donor (HD) PBMCs. Complete statistical analysis of the effect of KPL-404 and G28-5 on B-cells using one-way ANOVA on log transformed data with matched mixed-effects modeling for multiple comparisons tests of significance between different conditions. *p<0.05, **p<0.005.

Fig.S4

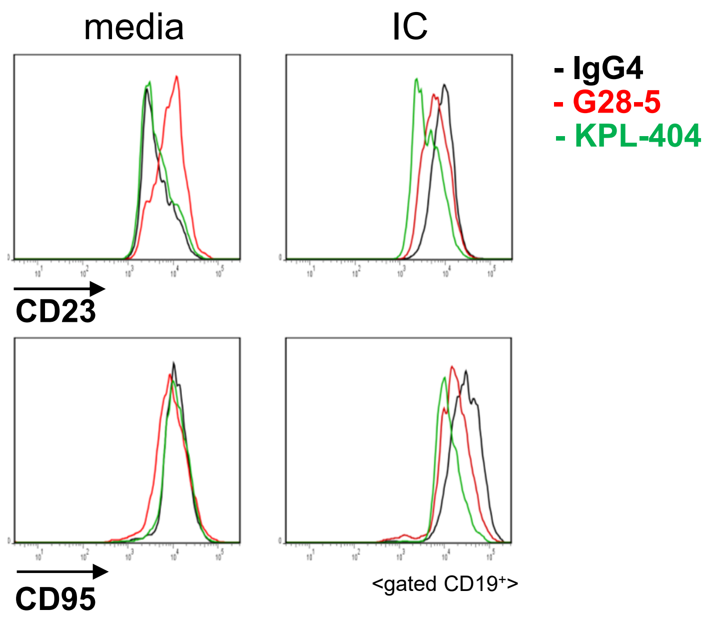

Fig.S4 Effects of KPL-404 and G28-5 on CD23 and CD95 expression on B cells. HD PBMCs were cultured in the presence of 10μg/ml IgG4 isotype control or anti-CD40 Abs KPL-404 or G28-5. Cells were left unstimulated (medium control), or stimulated with CD3/CD28 cross linker IC (18 hours in culture). Flow cytometry data, showing the levels of expression of CD23 and CD95 on gated $CD19^{+}$ B cells in each condition. Data is representative of two independent experiments.

Fig.S5

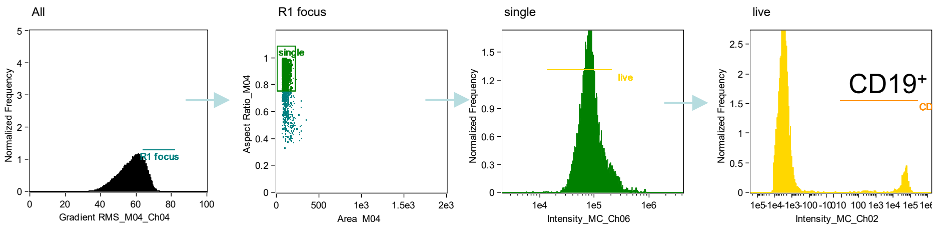

Fig.S5. Assessment of KPL-404 binding and internalization by Imaging flow cytometry – gating strategy. 2-5 million PBMCs were stained with anti-CD19-AlexaFluor (AL)488, anti-CD40 Abs KPL404-AL647 and G28-5-AL647, or, anti-CD22-AL647. Cells were either kept on ice, or incubated at 37°C for 1h, fixed in 1% formalin, and analyzed. Representative gating strategy used for the identification of CD19^{+} B cells; 400-500 B cell images were further analyzed for anti-CD40 Ab binding and internalization (as shown in **Fig.5)**.

Fig.S6.

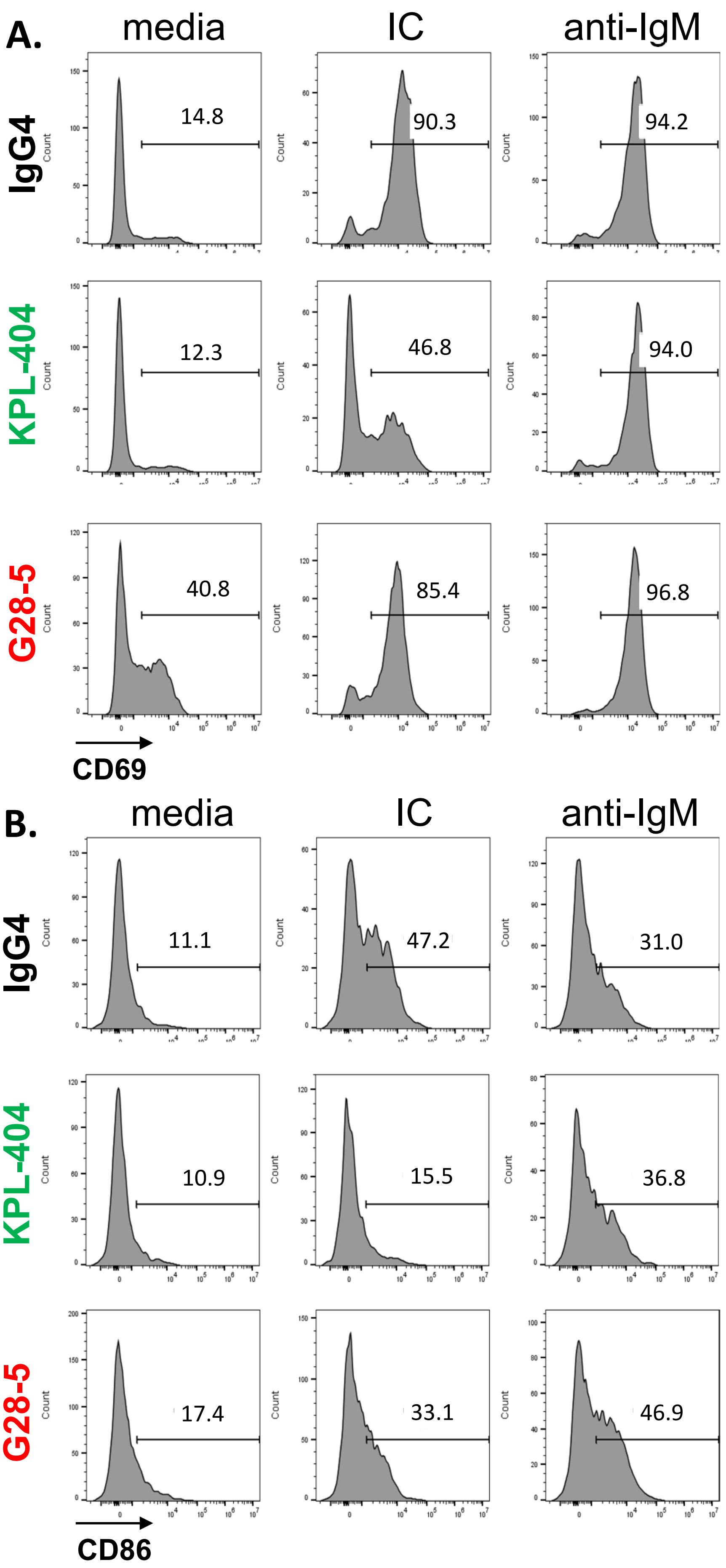

A.
media
IC
anti-IgM
IgG4
KPL-404
G28-5
14.8
90.3
94.2
12.3
46.8
94.0
40.8
85.4
96.8
CD69
B.
media
IC
anti-IgM
IgG4
KPL-404
G28-5
11.1
47.2
31.0
10.9
15.5
36.8
17.4
33.1
46.9
CD86
Count

C.

SjS

Treatment	CD69	CD86
IgG4+media vs IgG4+IC	*	**
IgG4+media vs KPL-404 +media	ns	ns
IgG4+media vs G28-5+media	ns	ns
IgG4+IC vs KPL-404+IC	*	ns
IgG4+IC vs G28-5+IC	ns	ns
IgG4+media vs IgG4+anti-IgM	*	ns
2C10+media vs KPL-404+anti-IgM	*	ns
KPL-404+media vs G28-5+anti-IgM	ns	ns
IgG4+IgM vs KPL-404 + anti-IgM	ns	ns
IgG4+IgM vs G28-5+ anti-IgM	ns	***

SLE

Treatment	CD69	CD86
IgG4+media vs IgG4+IC	***	***
IgG4+media vs KPL-404+media	ns	ns
IgG4+media vs G28-5+media	**	**
IgG4+IC vs KPL-404+IC	**	***
IgG4+IC vs G28-5+IC	ns	ns
IgG4+media vs IgG4+anti-IgM	***	*
KPL-404+media vs KPL-404+anti-IgM	***	**
G28-5+media vs G28-5+anti-IgM	***	ns
IgG4+IgM vs KPL-404+anti-IgM	ns	ns
IgG4+IgM vs G28-5+anti-IgM	ns	**

Fig.S6. Effects of KPL-404 on B cell activation in autoimmune patients assessed by the expression of the activation markers CD69 and CD86. PBMCs were cultured in the presence of 10μg/ml IgG4 isotype control or anti-CD40 Abs KPL-404 or G28-5 (16-18 hours of cell culture). Cells were left unstimulated (media control), or stimulated with CD3/CD28 cross linker IC, or $F(ab')_2$ goat anti-human IgM (anti-IgM) and B cell activation was assessed by the expression of the activation markers CD69 and CD86 on gated live $CD19^+$ B cells. **(A)** Representative data from one individual SLE patient, showing the expression of CD69**;** gates depict the frequencies of $CD69^+$ cells. **(B)** Representative data from one individual SLE patient, showing the expression of CD86**;** gates depict the frequencies of $CD86^+$ cells. **(C) Changes in the expression of CD69 and CD86 in SjS and SLE PBMC.** Complete statistical analysis of the effect of KPL-404 and G28-5 on B-cells using one-way ANOVA on log transformed data with matched mixed-effects modeling for multiple comparisons tests of significance between different conditions. *p<0.05, **p<0.005, and ***p<0.001.

Fig.S7

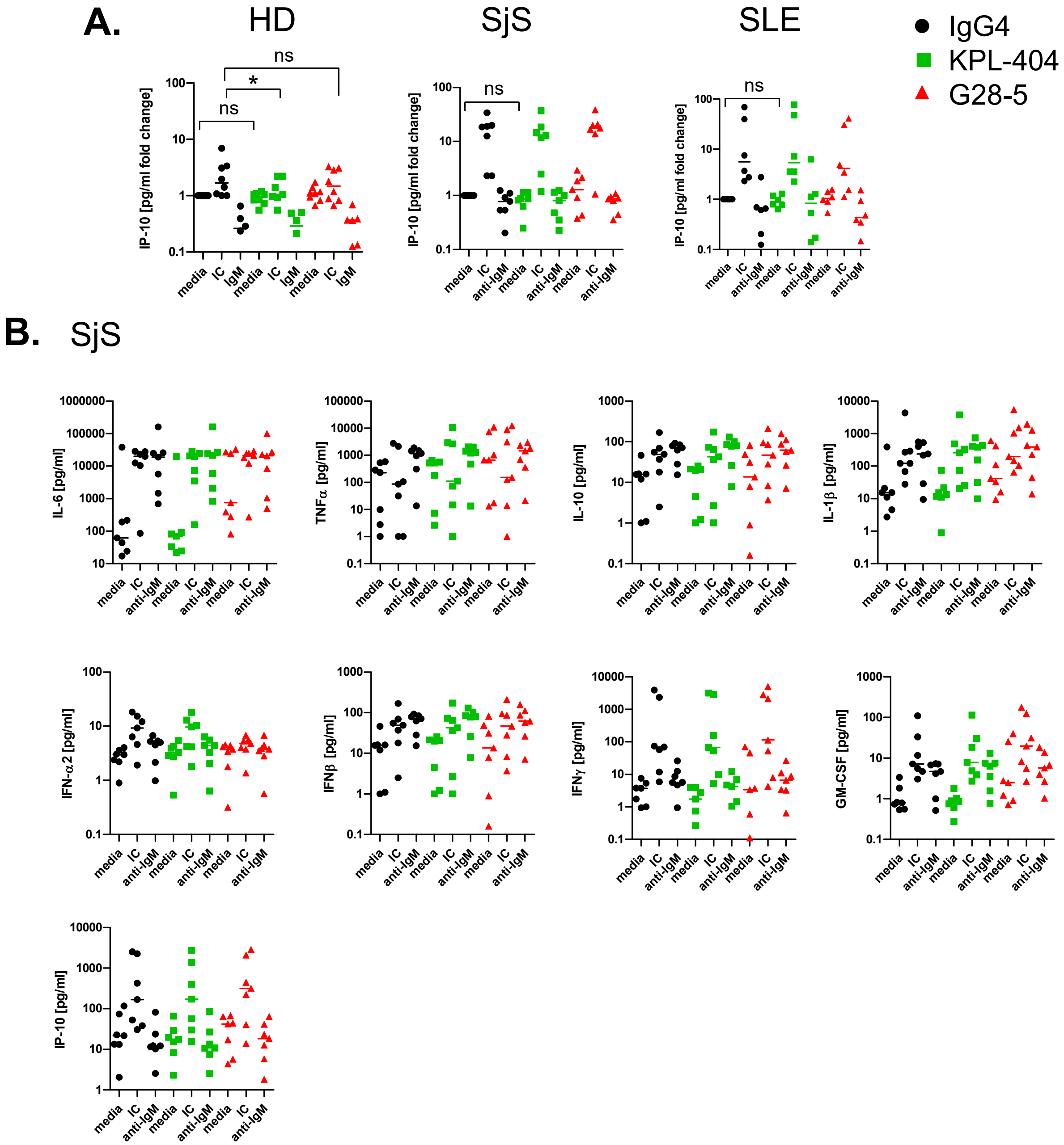

Fig.S7 Analysis of cytokine production in cell cultures (additional data). (A) Effects of KPL-404 and G28-5 on IP-10 production in PBMCs cultures from HD, SjS and SLE patients. The data for each sample were normalized against media controls and is expressed as a fold-change. Statistical analysis were performed using one-way Friedman multiple comparisons test and one-way ANOVA mixed effects analysis (for HD due to missing values); $^*p < 0.05$. **(B)** Cytokine production in SjS cell cultures presented as pg/ml.
